# Rheological Properties of Fish Gelatin Modified with Sodium Alginate

**DOI:** 10.3390/polym13050743

**Published:** 2021-02-27

**Authors:** Svetlana R. Derkach, Daria S. Kolotova, Nikolay G. Voron’ko, Ekaterina D. Obluchinskaya, Alexander Ya. Malkin

**Affiliations:** 1 Department of Chemistry, Murmansk State Technical University, 183010 Murmansk, Russia; derkachsr@mstu.edu.ru (S.R.D.); voronkong@mstu.edu.ru (N.G.V.); 2 Murmansk Marine Biological Institute of the Russian Academy of Sciences, 183010 Murmansk, Russia; obluchinskaya@mmbi.info; 3 A.V. Topchiev Institute of Petrochemical Synthesis, Russian Academy of Sciences, 119991 Moscow, Russia; alex_malkin@mig.phys.msu.ru

**Keywords:** fish gelatin, sodium alginate, polyelectrolyte complexes, gel–liquid transition, elastic modulus, creep, elastic recoil, viscoelastic media

## Abstract

Polyelectrolyte complexes of sodium alginate and gelatin obtained from cold-blooded fish were studied for potential application as structure-forming agents in food hydrogels. The mass ratio of sodium alginate to gelatin plays a decisive role in the sol-gel transition and rheological behavior of the complexes. Differences in the sol-gel transition temperature were observed upon heating and cooling, as is typical for such materials. We investigated the characteristics of this transition by measuring the isothermal changes in the elastic modulus over time at a constant frequency and the transition temperature at a range of frequencies. The kinetic nature of this transition depends on the composition of the complexes. A characteristic alginate-gelatin mass ratio is the ratio at which maximum transition temperature as well as elastic modulus and viscosity (rheological parameters) values are obtained; the characteristic mass ratio for these complexes was found to be 0.06. Calculation of the ionic group ratios in the biopolymers that form complexes and comparison of these data with the turbidimetric titration results clarified the origin of these maxima. Measuring the viscoelastic properties and the creep-elastic recoil of the samples allowed us to characterize these materials as viscoelastic media with a viscosity in the order of 10^3^–10^4^ Pa·s and an elastic modulus in the order of 10^2^–10^3^ Pa. These values drastically decrease at a certain stress threshold, which can be treated as the gel strength limit. Therefore, the observed rheological behavior of gels formed by fish gelatin modified with sodium alginate characterizes them as typical viscoelastic soft matter.

## 1. Introduction 

Gelatin, one of the most commonly utilized biopolymers, is widely used in the pharmaceutical, cosmetic, and food industries [1,2,3]. Gelatin has different biomedical applications in regenerative medicine and tissue engineering [4,5,6]. Due to the thermoreversible properties of its structure, gelatin provides a specific consistency to various products and allows the regulation of their organoleptic characteristics by modifying their rheological properties. Mammalian gelatin accounts for the vast majority of commercial gelatin [7,8], but its use has been subjected to constraints and criticism for many years. Fish gelatin, obtained from fish skin, scales, bones, and other fish parts, is considered an excellent alternative to mammalian gelatin [9,10] because of the similarity in the functional properties of the two types of gelatin [11]. Replacing mammalian gelatin with fish gelatin in the food industry would significantly expand the sales market by attracting those consumers for whom the consumption of products containing mammalian-derived gelatin is unacceptable for religious and ethnocultural reasons [2,8]. Furthermore, the use of fish gelatin would eliminate the risks of prion disease infection (in particular, spongiform encephalopathy) [8,12] and would partially solve the problem of waste disposal from the fish processing industry [13,14,15]. The total global fishing volume in 2018 was estimated at 179 million tons, and this figure increases yearly [16]. The waste from fish processing can account for 30% to 85% of the total catch, and a significant percentage of the waste (approximately 30%) comprises skin, bones, and scales, all of which have a high collagen content [14,16,17]. Therefore, the production and use of fish gelatin in food technology has numerous advantages.

Nevertheless, compared with mammalian gelatin, fish gelatin possesses inferior gelation and rheological properties, which limits its practical application. In particular, fish gelatin produces gels with considerably lower gelling and melting temperatures [18], storage moduli [19], and gel strengths [20,21] compared to mammalian gelatin gels. This is likely due to the lower proline and hydroxyproline content in fish gelatin compared to that of mammalian gelatin [22,23]. This is particularly true for gelatin obtained from cold-blooded fish [24,25,26]. One of the most promising strategies for improving the gelation properties of fish gelatin is via its modification with natural polysaccharides [27]. The introduction of gellan [28,29] or carrageenan [30] into the system improves the gelling properties of gelatin and the rheological properties of gelatin gels. One of the most commonly utilized compounds for this purpose is sodium alginate [31,32], an anionic polysaccharide obtained from brown seaweed. 

The interaction between gelatin and anionic polysaccharides leads to the formation of polyelectrolyte complexes of variable compositions. Complexes of gelatin with ionic polysaccharides are mainly formed via intermolecular electrostatic interactions and hydrogen bonding. Hydrophobic interactions are also involved, but they have smaller impacts on the complex formation [33,34]. The formation and stability of polyelectrolyte complexes depend on different factors including the degree of ionization of polyelectrolytes, the charge distribution over the polymeric chains, the nature and position of the ionic groups, the polymer chain flexibility, the molecular weight of the polyelectrolytes, the mixing ratio of the polyelectrolytes, as well as the temperature, ionic strength, and pH of a reaction medium [35].

At a certain biopolymer ratio, pH, and ionic power of the medium, these complexes become gels [36,37,38]. The numerous studies published on this subject have largely been focused on systems based on mammalian gelatin [39,40,41]. However, given that fish gelatin has a different secondary structure, amino acid composition, and rheological properties to that of mammalian gelatin, further research is required to better understand the exact mechanism of such modifications and expand the application of fish gelatin in the food industry. 

The goal of this study is to investigate complex formation between mixtures of cold-blooded fish gelatin and sodium alginate (SA) in varying ratios, evaluate the rheological properties of these systems, including the regularity of their gelation. The effect of the mass ratio of sodium alginate to gelatin on the characteristics of the sol-gel transition and the rheological properties of the resulting gels were studied.

## 2. Materials and Methods 

### 2.1. Materials

**Figure 1:**
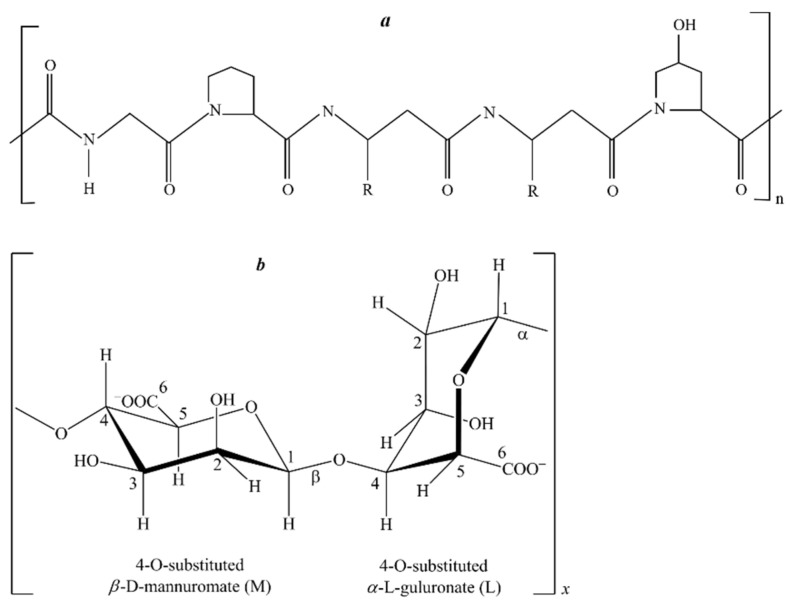
The structure of the gelatin (**a**) and sodium alginate (**b**) units.

Gelatin from the skin of cold-water fish (G 7041) was supplied by Sigma (Oakville, Ontario, Canada). The mass-average molecular weight (M_w_) was determined by high-performance liquid chromatography (HPLC) using an LCMS-QP8000 chromatography-mass spectrometer (Shimadzu, Kyoto, Japan) at λ = 280 nm. Chromatographic separation was performed using a Tosoh TSKgel Alpha-4000 column at 25 °C and a flow rate of 0.8 mL/min; the eluent was 0.15 M NaCl. The M_w_ of gelatin was 130 kDa. The structure of the gelatin unit is given in Figure 1a.

The isoelectric point (pI) was determined using a capillary glass viscometer VPZH-2 (Ekros, St. Petersburg, Russia) with a diameter of 0.34 mm at 25.0 ± 0.1 °C and by turbidimetric measurements using a T70 UV/visible spectrophotometer (PG Instruments, Midlands, UK) at 23.0 ± 0.5 °C. The pI of gelatin was 7.6.

The amino acid composition of the gelatin was determined by HPLC using an LC-10A liquid chromatograph (Shimadzu, Kyoto, Japan) with a SUPELCOSIL LC-18 column (4.0 mm × 30 cm, 5 μm) and the results are presented in Table 1. Gradient separation was performed with a binary eluent (acetonitrile/0.05 M sodium acetate solution) at a flow rate of 1.5 mL/min and a column temperature of 35 °C. The signals were recorded using a spectrofluorimetric detector (RF-10 AXL) with an excitation wavelength of 350 nm and an emission wavelength of 450 nm.

Proline and hydroxyproline content was determined using an LCMS-QP800a chromatograph-mass spectrometer (Shimadzu, Kyoto, Japan). The separation was performed using a SUPELCOSIL LC-18 column (4.0 mm × 25 cm, 5 μm) with an acetonitrile/ water mixture (volume ratio of water to acetonitrile 85:15) containing 0.01 M formic acid; the flow rate (eluent) was 0.75 mL/min, analysis time was 18 min, and sample volume was 10 μL (the gelatin was previously dissolved in a 0.05 M acetic acid solution).

Sodium alginate (SA) from brown algae (A2033, Sigma, Gillingham, United Kingdom) was used. The molecular mass distribution of the sodium alginate sample was determined by HPLC (Table 2). An LC-20A chromatograph equipped with an RID-10A refractive index detector (Shimadzu Corp., Kyoto, Japan) and Shodex Asahipak GS-520 HQ and GS-620 HQ (7.5 mm × 300 mm) columns was used. Columns were calibrated using standard pullulans with M_w_s of 6.2 to 740 kDa (P-82 kit, Showa-Denko, Japan) and blue dextran-2000 (Sigma-Aldrich). The molecular mass distribution of alginate was assessed by normalizing the peak areas [42]. The M_w_ of sodium alginate was determined as ~507 kDa. The structure of the sodium alginate molecule is given in Figure 1b.

Alginic acid content was determined by the color reaction using 3,5-dimethylphenol with sulfuric acid [43]. Absorbances of the standards and extracts were measured at 400 (A400) and 450 nm (A450). The alginic acid content of the samples was determined against the alginic acid standard at the effective absorbance of A400–A450. The alginic acid content of the sodium alginate sample was found to be 92.2% ± 0.7%.

### 2.2. Methods

#### 2.2.1. Sample Preparation

Aqueous solutions of gelatin and SA were prepared separately using the following protocol. In the first stage, samples of gelatin and SA were swelled in deionized water (18.2 MΩ·cm) at room temperature for 1 and 16 h, respectively. Then, the swollen samples were dissolved at 30 °C (for gelatin) and 70 °C (for SA). Then, mixtures of certain polymer concentrations were prepared and cooled to room temperature. Finally, samples with a gelatin concentration of 10 wt.% and SA concentration varying from 0.2 to 2 wt.% were prepared. Therefore, the mass ratio of SA to gelatin (Z = C_SA_/C_G_, g SA/g G) varied from 0.02 to 0.20. The pH values of the SA-gelatin mixtures were in the range of 5.2–5.9. In this pH range, phase separation or coacervation of the mixtures did not occur. 

Gelatin gels with or without sodium alginate were obtained by cooling the gelatin solution with or without aqueous polysaccharide to 4 °C for 24 h.

#### 2.2.2. Rheological Tests

The rheological properties of the samples were measured using an MCR 302 modular rheometer (Anton Paar, Graz, Austria) equipped with a cone-and-plate working pair. The diameter of the cone was 50 mm, the angle between the surface of the cone and plate was 1°, and the distance between the cone apex and the plate was 0.100 mm. The samples were stored at the required temperature for 24 h prior to the measurements.

The following experimental protocols were applied [44]:

- periodic oscillations with a constant frequency of ω = 6.28 rad/s, and amplitude sweep changes in the range of 0.01–1000%.
- periodic oscillations with a constant amplitude of γ = 1% (corresponding to the domain of linear viscoelasticity), and a frequency sweep in the range of 0.01–100 rad/s.
- temperature scanning with ramp rates of 0.5, 1 and 2 K/min at constant frequencies of 6.28 (1 Hz), 31.42 (5 Hz), 62.83 (10 Hz) and 125.66 rad/s (20 Hz) and a constant amplitude of deformation of γ = 1%;
- time dependence of the elastic modulus at temperatures ranging from 4 to 14 °C at a constant frequency of ω = 6.28 rad/s and constant amplitude of deformation of γ = 1% (to monitor the gelation kinetics), the initial temperature was 25 °C; the samples were sealed with silicone oil to prevent water evaporation;
- isothermal creep and elastic recovery at temperatures ranging from 4 to 10 °C and a constant stress in the range of 5–400 Pa for loading for 20 min and recovery for 20 min.

#### 2.2.3. Turbidimetric Measurements and UV-Spectroscopic Analysis

The interaction of gelatin with SA was investigated using turbidimetric titration. The gelatin solution (C_G_ = 0.25 wt.%) was titrated with a solution of sodium alginate (C_SA_ = 0.25 wt.%). The optical density was measured at a wavelength λ = 400 nm and optical path length l = 30 mm using a Unico 1201 spectrophotometer (Unico, Dayton, USA). The measurements were performed at room temperature (25 °C).

The ultraviolet (UV) absorption spectra of the gelatin solutions, SA solutions, and their mixtures were measured in the spectral range of 190–300 nm [45] using a T70 UV/visible spectrometer (PG Instruments, Midlands, United Kingdom) with an accuracy of 0.1 nm. The width of the quartz cuvette was 1 cm, and the measurement temperature was 25 °C.

## 3. Results

### 3.1. Temperature Transitions

For aqueous gelatin solutions, a decrease in the temperature leads to a sol-gel transition at a concentration above a certain threshold [46]. This critical concentration for cold-blooded fish gelatin is 10%, and the transition occurs at temperatures below 10 °C [26], while the critical concentration for mammalian gelatin is close to 1% at 15 °C [27]. The transition temperature can be increased by the addition of sodium alginate because SA-gelatin polyelectrolyte complexes can act as additional nodes in the structural network of the gel. 

**Figure 2:**
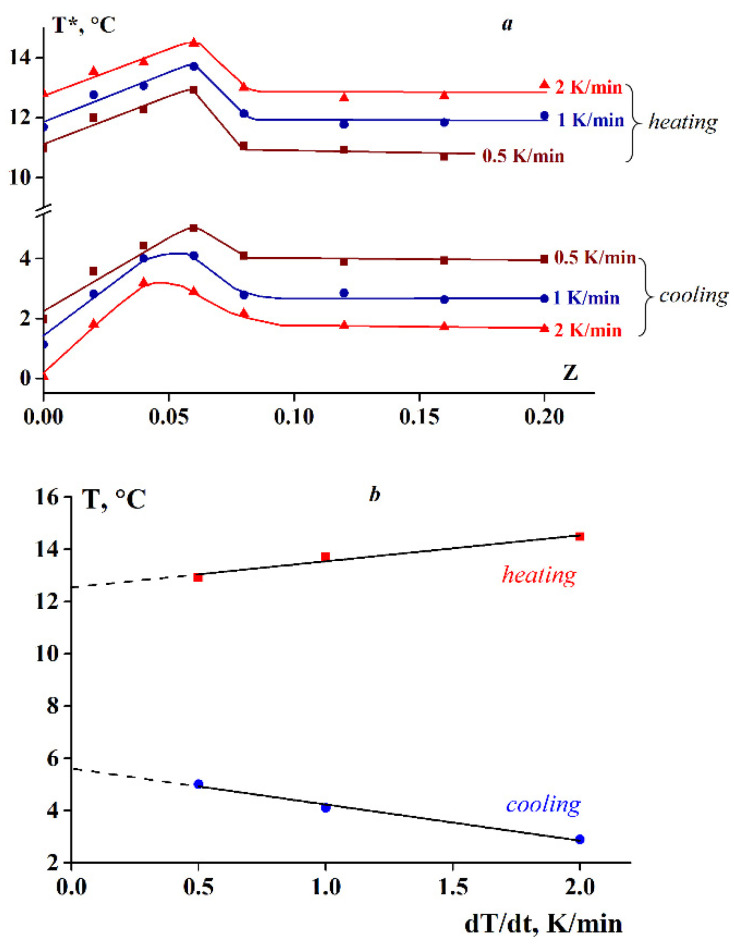
Transition temperature T* dependency on the SA-gelatin mass ratio Z for cooling and heating at temperature ramp rates shown on the curves (**a**) and transition temperature T* dependency on cooling and heating rates for the Z = 0.06 SA-gelatin mixture (**b**).

In this study, the influence of sodium alginate on the temperature of the sol-gel transition (T*) was investigated. The sol-gel transition temperature was determined by temperature scanning of the elastic moduli, namely the storage modulus (G’) and loss modulus (G′), at ramp rates ranging from 0.5 to 2 K/min during cooling/heating. In all cases, when measuring the elastic moduli, the same frequency was used (6.28 rad/s or 1 Hz), and the transition temperature, T*, was assumed as the crossover point (G’ = G′) [47]. The initial experimental data obtained by scanning at a constant temperature ramp rate for SA-gelatin mixtures with various Z values are presented in the Supplementary File (Figure S1). The results of these experiments are presented as the dependency of T* on Z for cooling and heating at three different temperature ramp rates (Figure 2a). 

Analysis of the experimental data indicated that the transition temperature is of a kinetic nature, as it depends on the composition of the SA-gelatin mixtures and the temperature scanning rate (Figure 2a). As shown in Figure 2a, the sol-gel transition temperature reaches a maximum at a certain ratio when Z = 0.06. As the temperature ramp rate increases, the transition temperature decreases in the cooling mode and, in contrast, increases in the heating mode. 

The rate dependence of the maximum T* values is shown in Figure 2b. Extrapolation to the static condition dT/dt = 0 (where t is time) does not exclude the difference in T* values obtained for heating and cooling. Of note here is the tendency toward a decrease in the difference between them.

**Figure 3:**
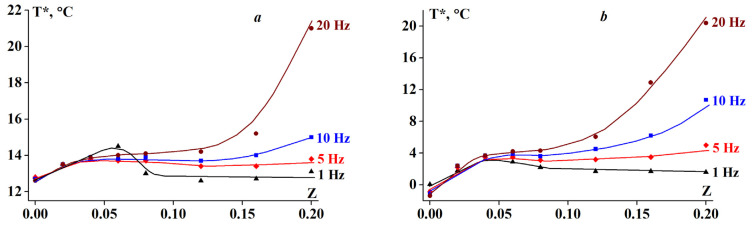
Transition temperature T* dependency on alginate-gelatin composition for (**a**) heating and (**b**) cooling at a temperature ramp rate of 2 K/min. G’, G′ (and T* as their crossover points) were obtained at the frequencies shown on the curves.

**Figure 4:**
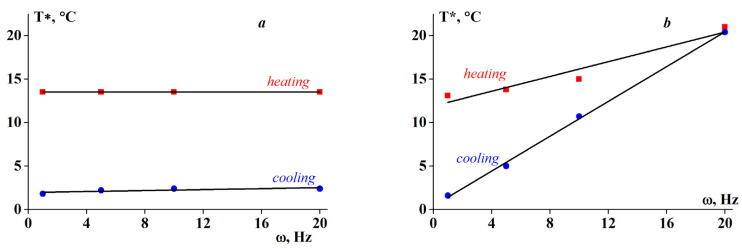
Transition temperature T* dependency on frequency for alginate-gelatin complexes with SA-gelatin mass ratios Z of (**a**) 0.02 and (**b**) 0.2 for heating and cooling.

The kinetic nature of the sol-gel transition is well illustrated by the data obtained at various frequencies. The original experimental data used to determine T* are included in the Supplementary File (Figure S2). Figure 3 and Figure 4 indicate that for systems with low SA content (Z < Z* = 0.06), the transition temperature T* is weakly dependent on the frequency. In contrast, at higher Z values, the role of frequency becomes more pronounced.

**Figure 5:**
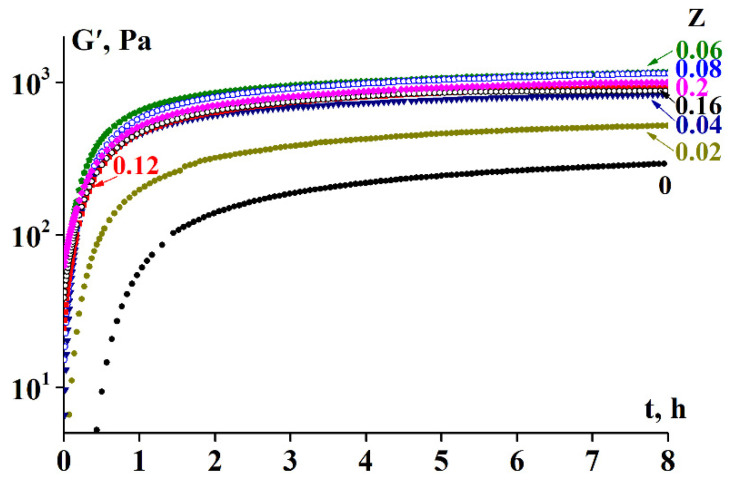
Gelation kinetics of SA-gelatin complexes at the Z values shown on the curves, T = 4 °C. (The initial temperature of the samples was 25 ° C), ω = 6.28 rad/s, γ = 1%.

As evident in Figure 5, the addition of the polysaccharide leads to a significant increase in the gelation rate, as evidenced by the rapid increase in the storage modulus G’. Thus, the characteristic gelation time (the time required to achieve constant values of G’) for gelatin without SA was approximately 8 h or longer [46], whereas for the SA-gelatin complexes, the gelation time was approximately 4–6 h. 

### 3.2. Rheology of Fish Gelatin Modified with Alginate

The majority of applications of SA-gelatin polyelectrolyte complexes are based on the use of these materials as gels. Therefore, measuring their rheological properties in the gel state is of primary interest.

**Figure 6:**
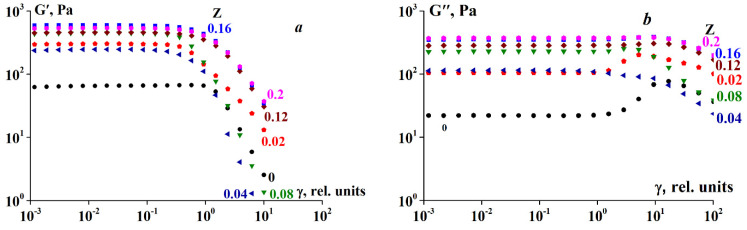
(**a**) Storage and (**b**) loss moduli dependency on amplitude at 4 °C. ω = 6.28 rad/s. Gelatin concentration 10 wt.%. Z values are shown on the curves.

An example illustrating the dependency of both elastic modulus components, the storage modulus G’ and loss modulus G′, on the amplitude for complexes with varying Z is presented in Figure 6.

Evidently, the storage modulus is dominant over the loss modulus for all compositions, formulated as G’ > G′. This is characteristic of a solid-like state of a material, and in this sense, the gel samples are considered as soft matter.

It has already been noted that the addition of SA, accompanied by complex formation, leads to an increase in gel rigidity, that is, higher Gʹ values (Figure 5). The same trend is observed in Figure 6. A second interesting observation is the relatively prolonged deformation range of linear behavior for all complexes. The limit of linearity for G’ exceeds γ values of 0.2–0.4.

Meanwhile, it is worth mentioning that the components of the elastic modulus presented in this figure at large amplitudes are not the ‘exact’ characteristics of the samples under study because the time dependency of the output signal (stress) at a given strain is not sinusoidal, and the theory for characterizing nonlinear viscoelasticity under large-amplitude oscillatory shear [48,49,50] should be applied to analyze non-harmonic signals. However, a discussion of the nonlinear effects is beyond the scope of this study.

**Figure 7:**
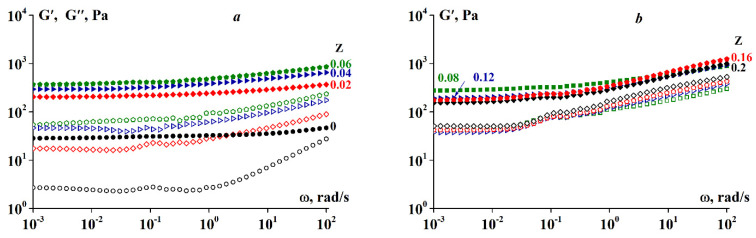
(**a**) G′ (closed symbols) and (**b**) G′′ (open symbols) moduli dependency on frequency at 4 °C at varying Z. Gelatin concentration was 10 wt.%. Z values are shown on the curves.

An example of the storage and loss moduli dependency on the frequency within the linear viscoelasticity range is shown in Figure 7. 

As can be seen in Figure 7a, in the frequency range of 10^−3^ to 10 rad/s, the storage modulus is virtually independent on the frequency for SA-gelatin polyelectrolyte complexes at Z < 0.06. Therefore, the storage modulus can be treated as the pseudo-equilibrium modulus G_pl_ [51]. Considering the complex as a gel-like network structure, one can estimate the apparent MW of a segment between neighboring entanglements, M_e_, according to the standard equation of the statistical theory for the elastic plateau:

(1) $$M_{e}\;=\;\frac{\mathrm{cRT}}{G_{\mathrm{pl}}}$$

where c is the concentration of the polymeric complex, R is the universal gas constant, and T is the temperature (K).

The G_pl_ values and, consequently, the Me values, lie within a relatively narrow range. For Z = 0.02–0.06, the range of G_pl_ is 207–382 Pa; then, R = 8.31 J/mol·K, and for C = 0.1 g/cm^3^, M_e_ is in the order of 10^6^ g/mol. Such high values indicate that the gel network is relatively sparse and weak. 

Figure 7b demonstrates that for SA-gelatin complexes at Z > 0.06, the storage modulus is constant only in a narrow range of low frequencies, showing a tendency to increase with increasing frequency. There is a distinct slope, implying that these gels are not entirely solids, but that superimposing viscous effects occur. 

**Figure 8:**
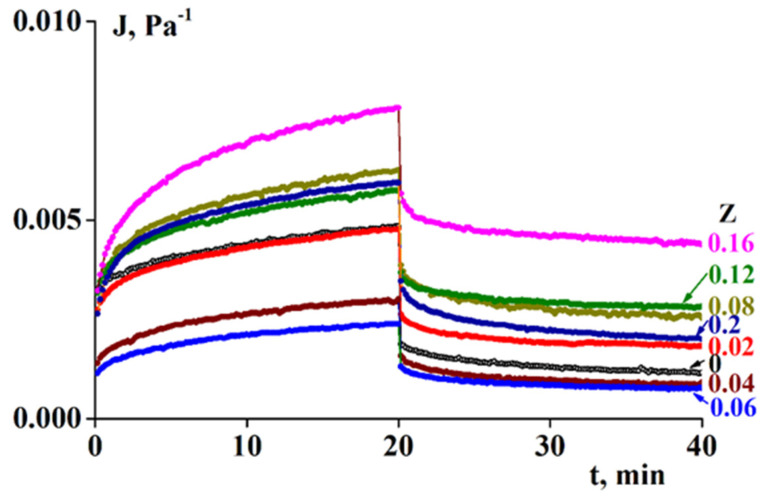
Compliance in loading (left side, t = 0–20 min) and recovery (right side, t = 20–40 min) with time. σ = 5 Pa, T = 4 °C.

To better understand the rheological properties of hydrogels formed by SA-gelatin complexes of various compositions, a series of experiments were performed in creep mode. Figure 8 presents a typical example of experimental data for creep and recovery at a constant temperature (4 °C) and constant stress (5 Pa). The full set of experimental data obtained for temperatures in the range of 4–15 °C and varying stresses (5–500 Pa) are presented in the Supplementary File (Figures S4 and S5).

The initial experimental data are presented as compliance versus time for shear compliance loading and unloading, where compliance, J, is the ratio of time-dependent deformations at a given stress:

(2) $$J(t,\;\sigma )\;=\;\frac{\gamma (t)\;}{\sigma }{=\;G}^{-1}$$

The principal conclusion that follows from the experimental data is that in almost all cases, there is a combination of reversible (elastic, γ_el_) and irreversible (flow, γ_fl_) components in complete deformation. This allows us to discuss the experimental data in terms of the elastic modulus G, defined as:

(3) $$G\;=\;\frac{\sigma }{\gamma_{\mathrm{el}}}$$

and shear viscosity η, defined as:

(4) $$\eta \;=\;\frac{1}{\mathrm{dJ}/\mathrm{dt}}\;=\;\frac{1}{{d\gamma }_{\mathrm{fl}}/\mathrm{dt}}$$

where γ_el_ is the residual deformation corresponding to the height of the plateau on the right-hand portion of J(t) dependence, and dγ_fl_/dt is determined by the steady slope in the left-hand portion of J(t) dependence.

**Figure 9:**
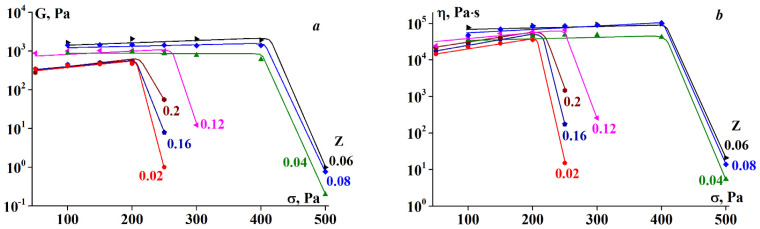
Dependence of (**a**) elastic modulus and (**b**) shear viscosity on shear stress at T = 4 °C. Z values are shown on the curves.

The elastic modulus and apparent viscosity as functions of shear stress are plotted in Figure 9. Both rheological parameters remain virtually constant in a certain stress range, demonstrating a certain pattern. The stress range wherein G and η are constant increases with increasing Z until the characteristic value of Z* = 0.06, whereupon it decreases for gels of complexes with Z > Z*. The same was true for the elastic modulus and viscosity. As shown in Figure 9, the highest elastic modulus and viscosity values were observed for gels at an alginate-gelatin mass ratio of Z = Z*.

The drastic decrease in the elastic modulus and viscosity at a certain threshold stress, shown in Figure 9, can be explained by gel rupture, treating this stress as the strength limit of the material or less likely, as a separation of the sample from a solid boundary surface, and transition to slip. Therefore, the elastic modulus and viscosity values presented in Figure 9 at stresses exceeding this limit should not be treated as the real physical parameters of the material.

Upon heating, the gel-to-sol transition should be accompanied by a change in the rheological properties of the entire complex. This is a transition from a (mild) solid body to a liquid. This transition is usually associated with a loss of elasticity and the start of flow. This effect can be monitored by measuring the evolution of viscoelastic properties in the ratio of elastic and plastic components of deformations in creep.

**Figure 10:**
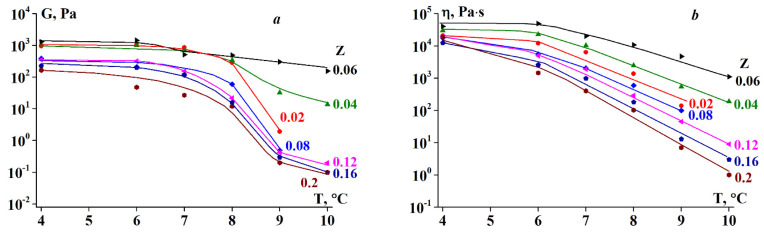
(**a**) Elastic modulus and (**b**) shear viscosity dependency on temperature at σ = 5 Pa. Z values are shown on the curves.

Figure 10 shows the elastic modulus and viscosity dependency on temperature in the range of 4–10 °C. It can be seen that the gels are relatively stable in this temperature range, although the elastic modulus and viscosity decrease with increasing temperature. 

### 3.3. Composition of Gelatin/Sodium Alginate Systems

**Figure 11:**
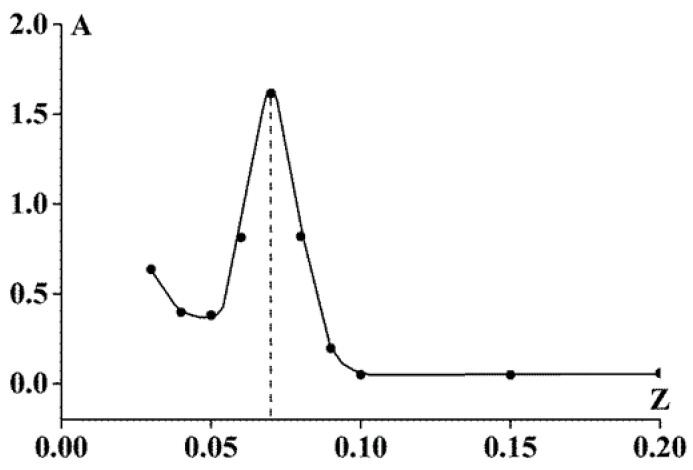
Optical density as a function of SA-gelatin mass ratio in the titration of gelatin sol (C_G_ = 0.25%) with SA sol (C_SA_ = 0.25%); λ = 400 nm, l = 1 cm, 23 °C.

In this study, the complexes were characterized to rationalize the rheological behavior of the systems under study. The biopolymer mass ratio, corresponding to the boundary of stoichiometric polyelectrolyte complex formation, was determined by turbidimetric titration. According to the classic concept developed in [52,53,54], during the formation of stoichiometric complexes, the negative charges of sodium alginate (host-polyelectrolyte) are entirely screened by the positive charges of gelatin (guest-polyelectrolyte). The turbidimetric titration results are shown in Figure 11. The increase in the optical density arises from an increase in the content of stoichiometric non-soluble SA-gelatin complexes. These complexes exist in the system, together with unbound gelatin macromolecules. 

The observed maximum at Z ≅ 0.07 on the curve corresponds to the formation of the maximum number of stoichiometric complexes as a result of the mutual neutralization of chain charges and the most effective ion pair formation [55]. A further increase in Z leads to a decrease in the optical density of the mixtures owing to the formation of soluble non-stoichiometric SA-gelatin complexes of variable composition. As SA content increases, the SA negative charge becomes uncompensated, leading to an increase in the solubility of the complexes and a decrease in the optical density.

This result is supported by the following estimations. At Z = 0.07, the gelatin-to-alginate weight ratio is 14.3, and the gelatin-to-alginate MW ratio is 0.25. Therefore, the molecular ratio of the components can be taken as 57. Thus, according to these estimates, one SA molecule can bind a maximum of 57 gelatin molecules. 

**Figure 12:**
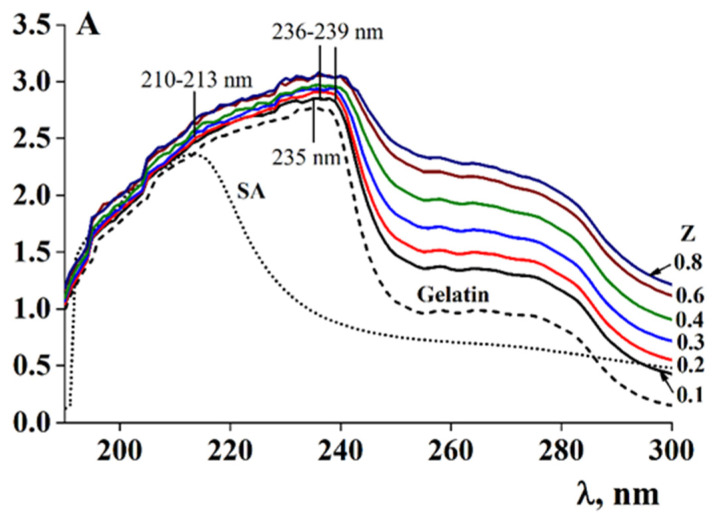
UV absorption spectra for sodium alginate (C_SA_ = 0.5 wt.%), gelatin (C_G_ = 1.0 wt.%) and aqueous mixtures of SA-gelatin mixtures with varying mass ratios Z.

The UV absorption spectra of solutions of SA, fish gelatin, and SA-gelatin mixtures with biopolymer mass ratios Z ranging from 0.1 to 0.8 are given in Figure 12. In the SA solution, maximum absorption was observed at λ = 213 nm. This is related to the presence of chromophores, such as hydroxyl and carboxyl groups, in the polysaccharide molecule, which absorb in the ultraviolet range [45]. 

A wide absorption band was detected for the gelatin solution at λ = 235 nm (Figure 12). Undivided nitrogen electron pairs conjugated with the double bonds in histidine and arginine residues [45], as well as conjugated double bonds in the benzene nuclei of aromatic amino acids, particularly tyrosine, [56] contribute significantly to the position of the gelatin absorption band.

It was demonstrated that the introduction of SA into the gelatin solution led to a bathochromic shift of the maximum adsorption from 236 to 239 nm, accompanied by an increase in the optical density and a significant broadening of the resulting absorption band (Figure 12). The observed changes in the gelatin spectra are attributed to electrostatic interactions between the charged carboxyl groups of the residues of β-D-mannuronic and a-L-guluronic acids of alginate, and the histidine, arginine, and lysine amino groups of gelatin. These changes also arise from hydrogen bonding between the hydroxyl groups of SA and the tyrosine residues of gelatin. An increase in absorption in this spectral region is related to light scattering by the relatively large particles of the biopolymer complexes.

## 4. Discussion

The structure and properties of the SA-gelatin complex gels are dependent on the conditions of their formation and temperature [57]. The difference in sol-gel transition temperature (T*) obtained for heating and cooling (Figure 2) is associated with the kinetic nature of the process. Initially, the gels subjected to heating were created under isothermal conditions at 4 °C for 1 d; such gels were more thermostable than gels obtained under continuous cooling. Prolonged structure formation leads to an increase in the number of triple helices, and thus in the density of the junction zones [58,59]. As this is a kinetic process, molten gels are prone to supercooling and delayed formation of triple helix junction zones. Consequently, the transition temperature depends on the heating/cooling rate. Therefore, gel thermostability can be enhanced by reducing the cooling rate and prolonging storage at low temperatures. These experimental results correlate with the general tendencies documented for various gelatin–polysaccharide complexes [60,61,62]. 

The two-part analysis in Figure 4 shows the effect of frequency on the transition temperature, being more prominent for systems with a high Z (Figure 4b). Different frequencies show different types of molecular motion. It can be assumed that both gelation and gel melting processes do not depend on the frequency for homogeneous systems (low Z), but become sensitive at high Z due to the inhomogeneity of the systems (high Z).

As discussed above, the sol-gel transition in the gelatin system and its SA mixture is a kinetic process occurring over time and therefore depends on the scanning rate. The kinetic nature of the transition process is also illustrated by the storage modulus dependency on time. A typical example at a temperature of 4 °C is presented in Figure 5, while the complete experimental data set is provided in the Supplementary File (Figure S3).

Analysis of the experimental data indicated differences in the behavior of systems with low and those with high SA to gelatin mass ratios (Z) during gelation (Figure 2, Figure 3 and Figure 5). The Z-boundary-value separating systems of distinct behavior is approximately 0.06, at which the maximum T* was obtained (Figure 2). These differences are possibly due to the differences in the spatial structures of the systems depending on Z, which in turn is associated with the stoichiometric relationship or its absence for active groups in polysaccharide and gelatin molecules. To verify this assumption, further experiments were performed to determine the composition of the SA-gelatin polyelectrolyte complexes.

Considering the composition and spatial structure of the SA-gelatin systems, determined by turbidimetric titration and UV spectroscopy (see Section 3.3), the following conclusions can be drawn: At low alginate-gelatin mass ratios, less than the characteristic value, Z ≤ Z* = 0.06 (0.07), the structure of the system is homogeneous; it consists of stoichiometric SA-gelatin complexes and unbound gelatin macromolecules. At larger mass ratio values, Z > Z*, the system is characterized by a heterogeneous structure, arising from the presence of non-stoichiometric complexes, the composition of which varies with (increasing) Z. This leads to the distinct behavior between systems with low and high Z (Figure 2, Figure 3 and Figure 5). Changes in the internal structure of the system should affect the rheological properties of the complex gels.

**Figure 13:**
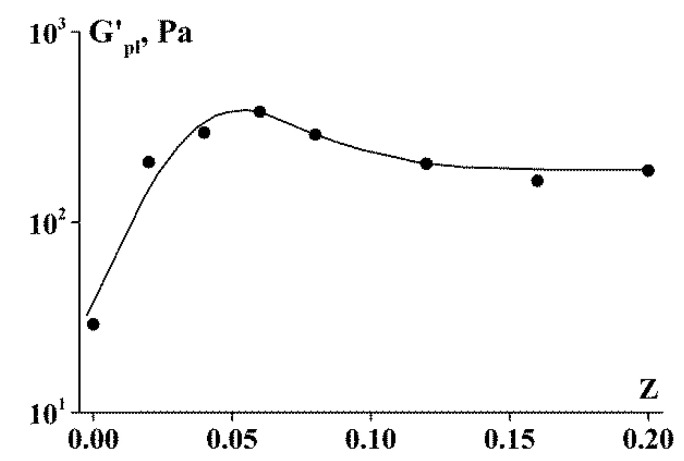
Storage modulus dependency on Z.

**Figure 14:**
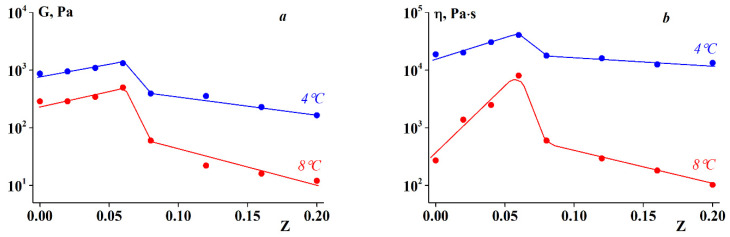
(**a**) Elastic modulus and (**b**) shear viscosity dependency on Z at σ = 5 Pa (elastic and plastic components of deformations in creep). Temperature values are shown on the curves.

The experimental data (Figure 10 and Figure 14) show the distinct rheological behaviors of the two types of SA-gelatin complexes. An increase in the concentration of stoichiometric complexes of the same composition for Z values below 0.06 (see Section 3.3) leads to an increase in the viscoelastic characteristics of the gel, which is due to the formation of additional junction zones in the gel network. The rheological parameters were optimal (Figure 13 and Figure 14) at the highest concentration of stoichiometric complexes, attained at Z = 0.06. The appearance of soluble non-stoichiometric complexes of variable compositions at Z > 0.06, and consequently, the appearance of an inhomogeneous structure (see Section 3.3) causes a decrease in the viscoelastic characteristics of the gel. The decisive roles of SA-gelatin-complex composition and the internal structure of the system are also illustrated by changes in the sol-gel transition temperature (see Section 3.1).

Considering these data as well as numerous similar results obtained in studies of viscoplastic properties of protein-polysaccharide complexes [63,64,65,66], it is worth discussing the concept of ‘gel’, often used to describe these products. The formal definition of a gel treats this object as non-flowing and, therefore, it is considered a soft, solid-like elastic material. Meanwhile, in most cases, we encounter the superposition of flow and elasticity; thus it is useful to make some comments concerning the terminology.

It is necessary to separate the terms ‘gel state’ and ‘gel-like behavior. The first case corresponds to the standard understanding of a gel as a soft solid material. However, gel-like behavior implies that there is a domain of small stresses and deformations where a material behaves as a soft solid, but at sufficiently high stresses, large deformations, and prolonged loading, the material begins to flow. This is a well-known phenomenon observed when a gel article that has been standing on a plate for a long time begins to sag and/or release liquid. The same can occur when one presses a gel-like product. In all of these cases, we encounter the superposition of solid-like and liquid-like behavior. In the experiments described above, this type of behavior is illustrated in Figure 6, wherein G″ becomes slightly higher than G at large deformations, and it is also reflected in Figure 10, wherein a direct superposition of elastic deformations and flow is observed. Numerous examples of such rheological behavior of the materials studied herein are provided in the Supplementary File.

SA-gelatin stoichiometric complexes obtained at Z ≤ Z* = 0.06 can potentially be used to create food products with certain rheological and organoleptic characteristics. Gelatin-containing products should be stored at temperatures of approximately 0–8 °C under conditions favorable for arresting the growth rate of microorganisms to prolong their storage life [67]. This temperature regime can also maintain the organoleptic and structural properties of food products, for example, culinary products.

## 5. Conclusions

A systematic study of the rheological properties of gels formed by polyelectrolyte complexes comprising cold-water fish gelatin and SA in a wide range of mass ratios was conducted. The results indicated that the sol-gel transition is a kinetic process, although gel formation occurs relatively rapidly at low temperatures. The kinetic characteristics of the transition temperatures for cooling and heating are distinct and depend on the composition of the complexes.

The maximum sol-gel transition temperature and maximum elastic modulus and viscosity (viscoelastic parameters) values of the complex gels were observed at a characteristic mass ratio of SA to gelatin Z* = 0.06 (0.07), and this ratio quantitatively correlates with the concentration of ionic groups in the components of the complexes. This boundary-value Z* separates systems with distinct internal structures and, consequently, distinct rheological behavior. The homogeneous structure of the systems formed by stoichiometric SA-gelatin complexes at Z < Z* is characterized by an increase in the elastic modulus with an increase in SA concentration. In contrast, inhomogeneous structures formed by water-soluble non-stoichiometric complexes of variable composition at Z > Z* cause a decrease in the viscoelastic characteristics of the gels with an increase in polysaccharide concentration. 

Measuring viscoelastic properties at varying frequencies and creep-recovery behavior allowed us to classify the objects under study as viscoplastic soft matter with a viscosity in the order of 10^3^–10^4^ Pa·s and an elastic modulus in the order of 10^2^–10^3^ Pa. A weak random network exists in these complex gels. At a certain stress threshold, the network is degraded. 

The use of fish gelatin modified with sodium alginate in the food industry is a safer alternative to mammalian gelatin, and can attract potential consumers for whom the consumption of products containing mammalian gelatin is unacceptable for religious and ethnocultural reasons. Furthermore, the production and widespread use of modified fish gelatin can partially address the issue of excessive waste from the fish processing industry.

## Figures and Tables

**Table 1:** Amino acid composition of gelatin from cold-water fish skin.

Amino Acid	Amount (g/100 g)	Amino Acid	Amount (g/100 g)
Glycine	18.6	Alanine	9.4
Proline	12.9	Taurine	2.9
Hydroxyproline	9.6	Tyrosine	0.8
Aspartic acid	5.6	Valine	2.1
Glutamic acid	9.3	Methionine	1.6
Serine	6.7	Isoleucine	1.5
Histidine	1.7	Leucine	2.8
Threonine	2.6	Lysine	2.3
Arginine	7.6	Phenylalanine	2.4

**Table 2:** Molecular weight distribution of sodium alginate.

Peak Number	M_n_, kDa	M_w_, kDa	Peak Area, %
1	839–975	990–1150	32–34%
2	523–540	610–630	38–40%
3	175–193	200–220	5.5–7.5%
4	89–143	100–160	2–14%

## Data Availability

The data presented in this study are available on request from the corresponding author.

## Supplementary Materials

The following are available online at https://www.mdpi.com/2073-4360/13/5/743/s1, Figure S1: Measuring temperature dependencies of the components of the dynamic modulus for complexes of different compositions—determination of gel/liquid transition by the crossover of the G’ and G” dependencies, Figure S2: Determination of the transition temperatures at the constant ramp of the temperature scanning at different frequencies, Figure S3: Kinetics of gelation at different temperatures for complexes of different compositions, Figure S4: Determination of the viscoelastic properties of complexes with different compositions in the creep-elastic recoil experiments, Figure S5: Creep and recovery curves obtained at different stresses.
