# Supplementary material for: Rheological Properties of Fish Gelatin Modified with Sodium Alginate
Source: Polymers (Basel). 2021 Feb 27;13(5):743. doi: 10.3390/polym13050743 (PMC7957763; doi:10.3390/polym13050743)
Supplement: Supplementary file 1 [file polymers-13-00743-s001.pdf]

Supplementary information

S1. Measuring temperature dependencies of the components of the dynamic modulus for complexes of different compositions – determination of gel/liquid transition by the crossover the G' and G" dependencies

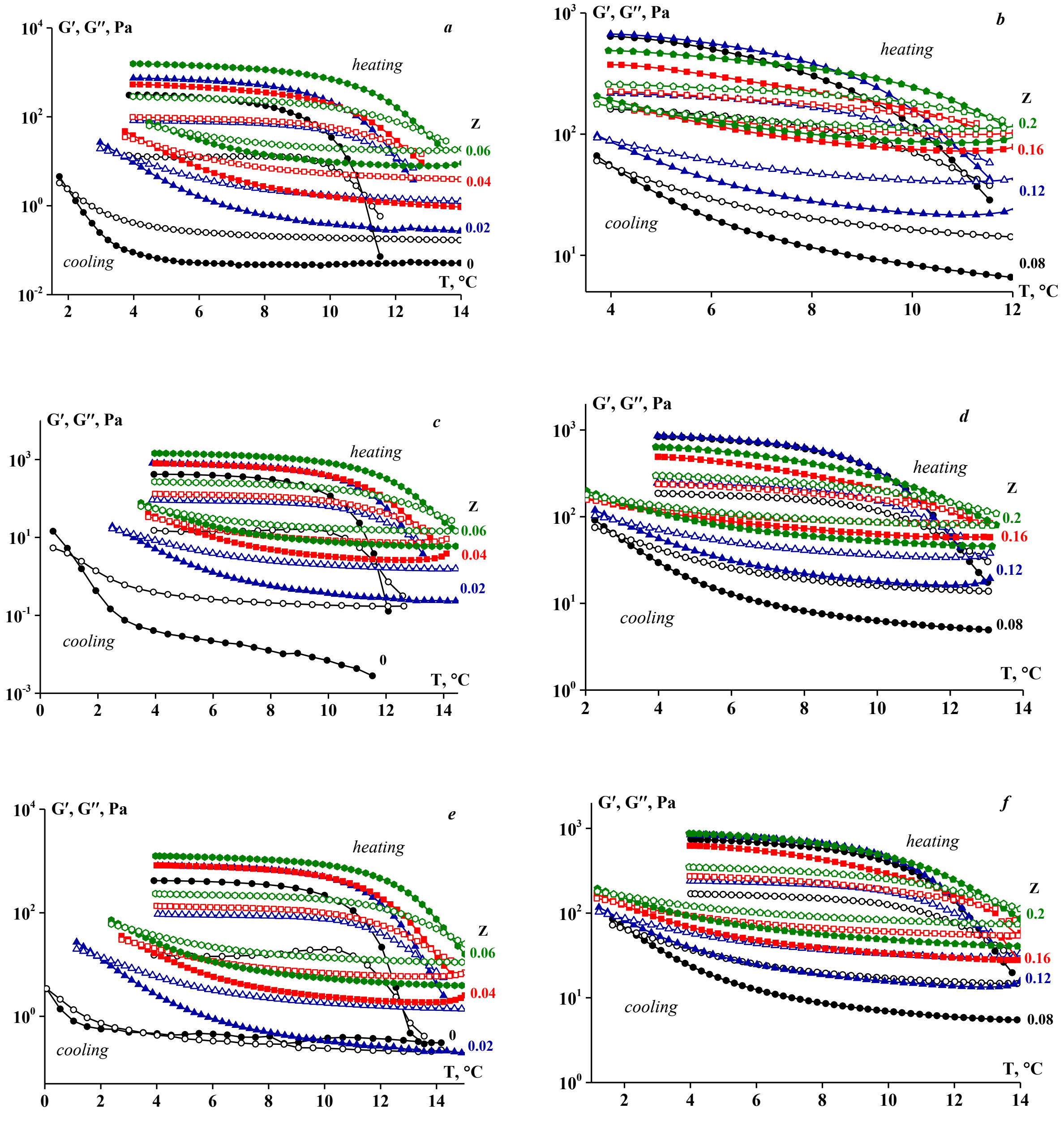

Fig. S1. Storage (closed symbols) and loss moduli (open symbols) as a function of temperature at γ = 1 % and ω = 6.28 rad/s for the complexes of different concentrations Z and the cooling-heating measurements at the constant ramp of the temperature scanning (K/min): 0.5 (a, b), 1(c, d), 2 (e, f).

S2. Determination of the transition temperatures at the constant ramp of the temperature scanning at different frequencies

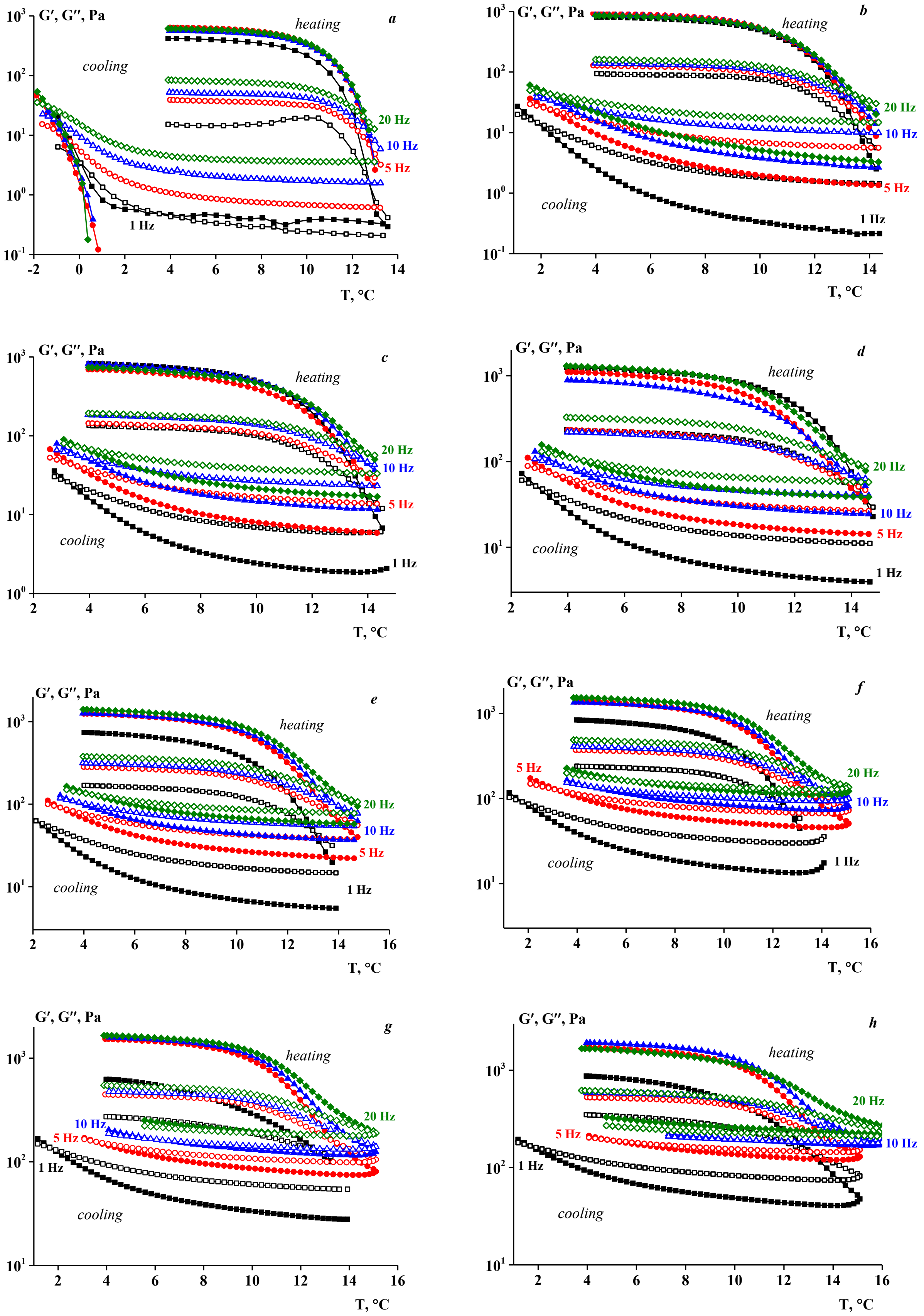

Fig. S2. Storage (closed symbols) and loss moduli (open symbols) as a function of temperature at the constant ramp of the temperature scanning (2 K/min) at different frequencies. Z: a – 0; b – 0.02; c – 0.04; d – 0.06; e – 0.08; f – 0.12; g – 0.16; h – 0.2. $\gamma = 1$ %.

S3. Kinetics of gelation at different temperatures for complexes of different compositions

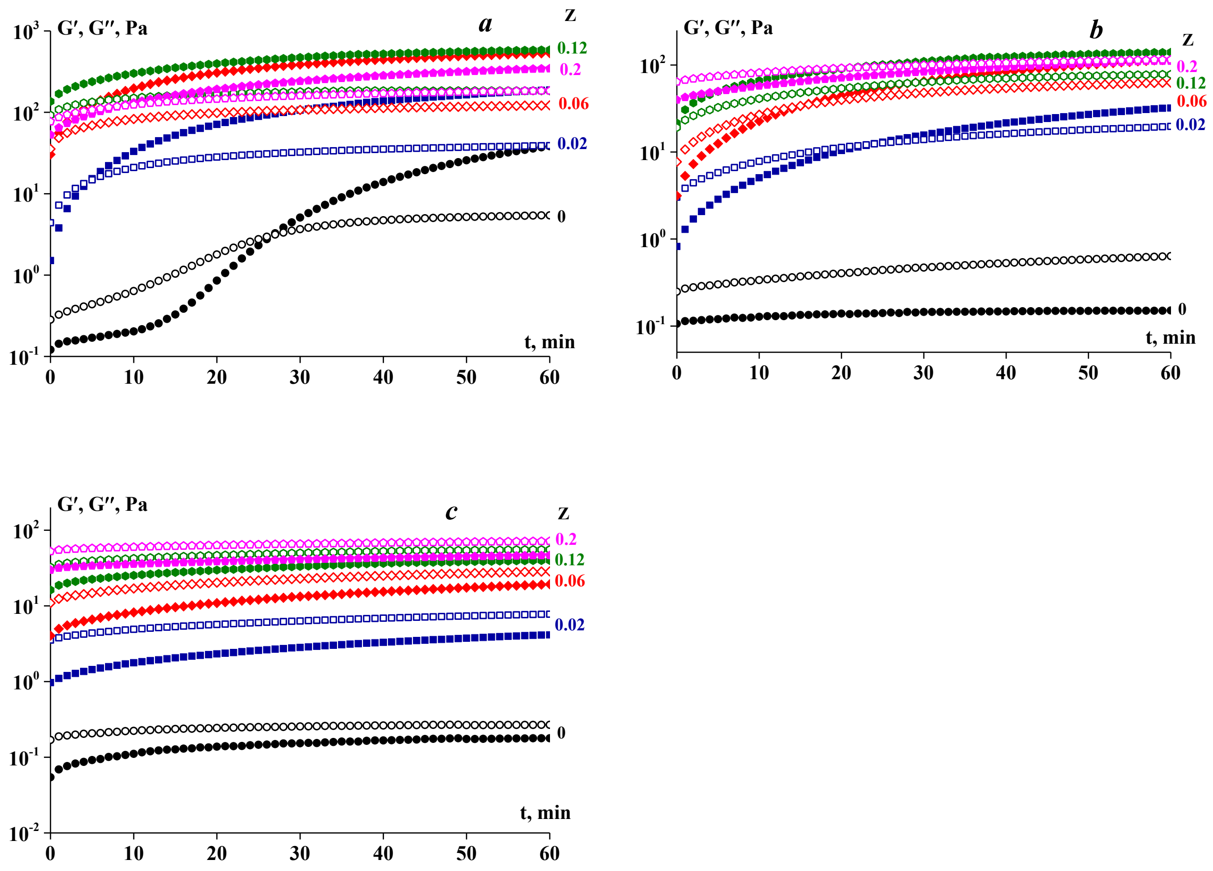

Fig. S3. Storage (closed symbols) and loss moduli (open symbols) of the SA-gelatin complexes as a function of time at different temperatures (T, °C): a – 4, b – 6, c – 8. $\gamma = 1$ %, $\omega = 6.28$ rad/s.

S4. Determination of the visco-elastic properties of complexes with different compositions in the creep-elastic recoil experiments

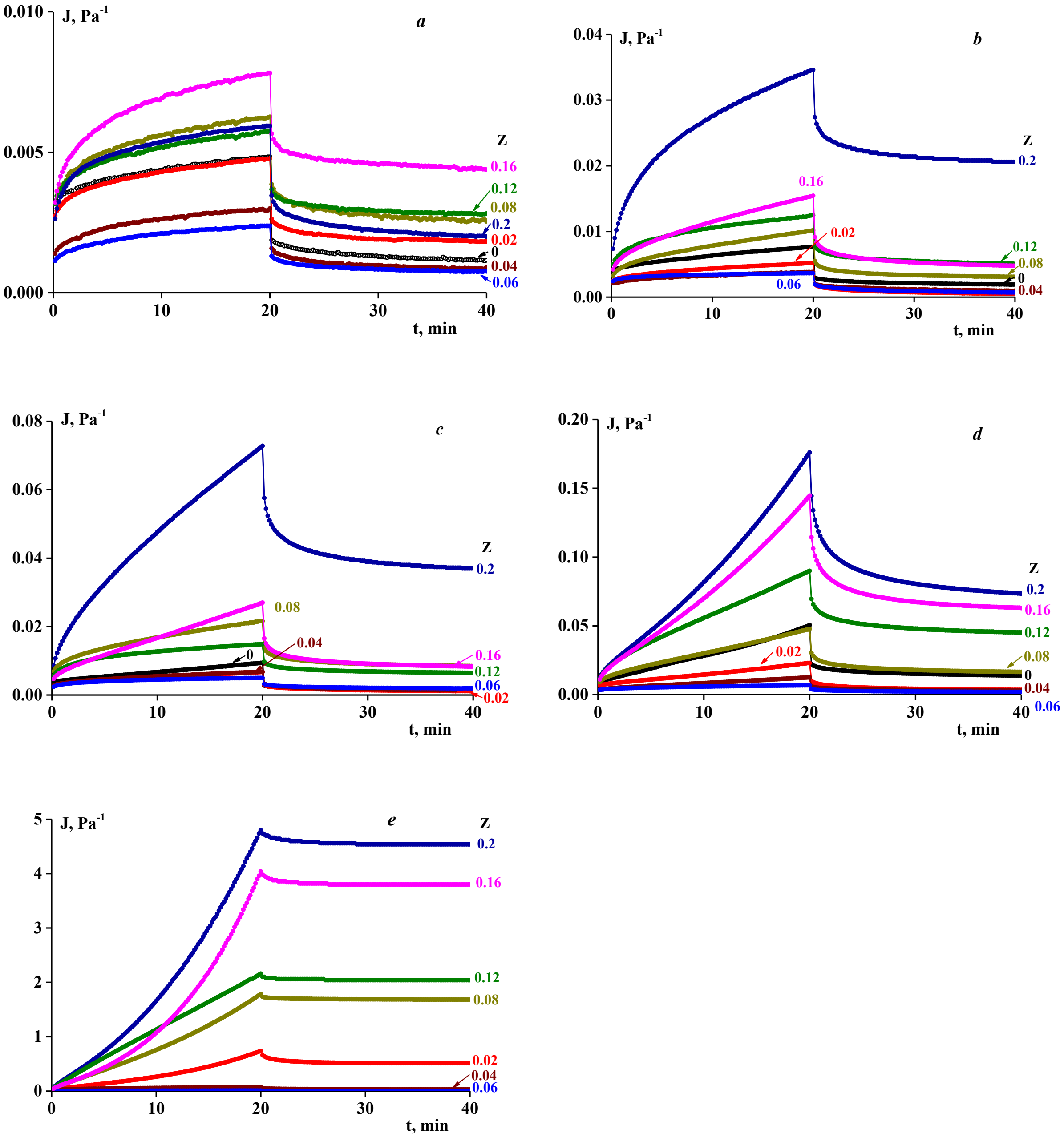

Fig. S4. Creep and recovery curves obtained at different temperatures (T, °C): a – 4, b – 6, c – 7, d – 8, e – 9. σ = 5 Pa.

S5. Creep and recovery curves obtained at different stresses

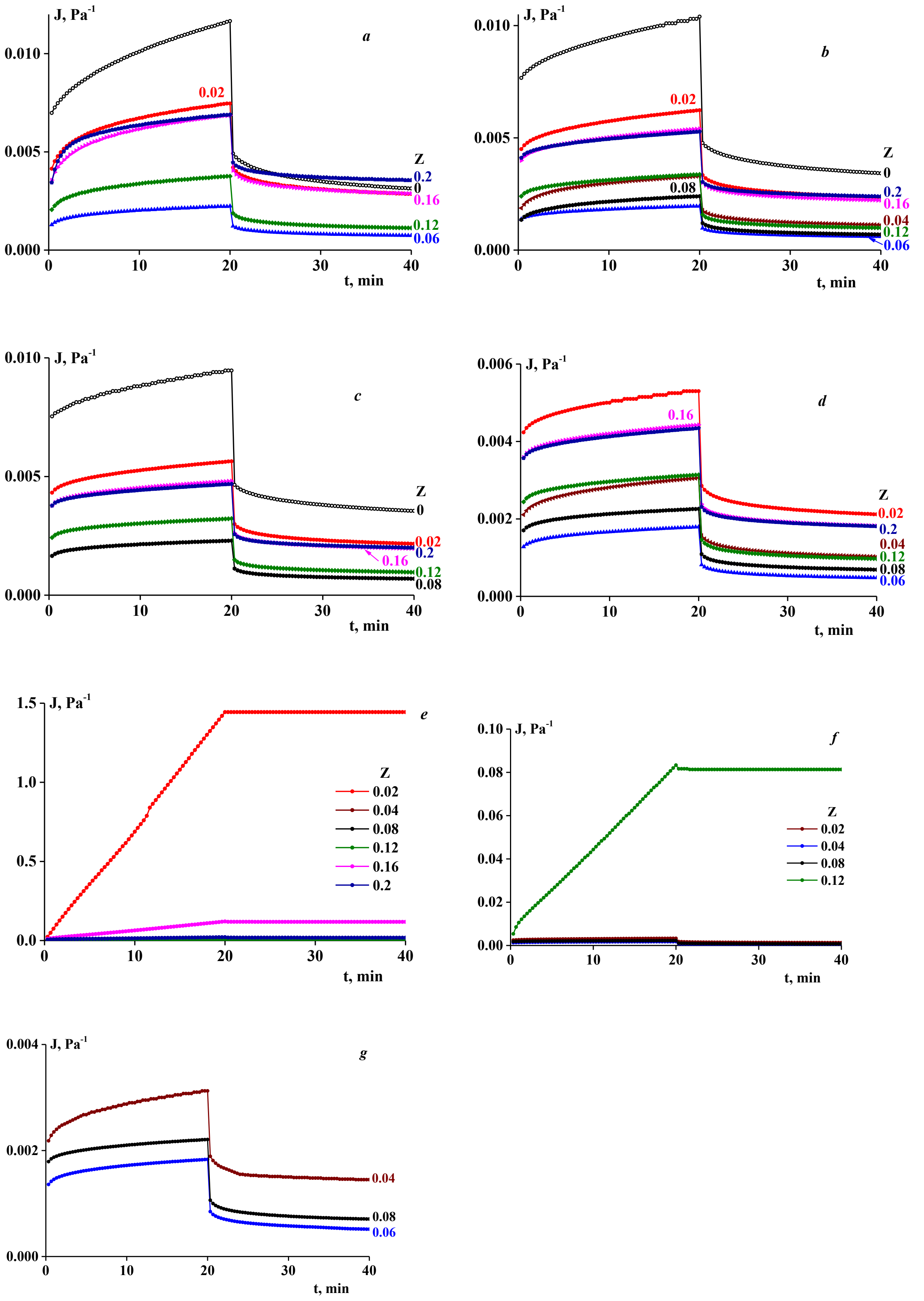

Fig. S5. Creep and recovery curves obtained at different stresses (σ, Pa): a – 50, b – 100, c – 150, d – 200, e – 250, f – 300, g – 400. T = 4 °C.
